# Chimeric bifunctional oligonucleotides as a novel tool to invade telomerase assembly

**DOI:** 10.1093/nar/gku688

**Published:** 2014-07-31

**Authors:** Dulat Azhibek, Maria Zvereva, Timofei Zatsepin, Maria Rubtsova, Olga Dontsova

**Affiliations:** 1 Department of Chemistry and A.N. Belozersky Institute of Physico-Chemical Biology, Moscow State University, Moscow, 119992, Russian Federation; 2 Skolkovo Institute of Science and Technology, Novaya Street, 100, Skolkovo, Odintsovsky District, Moscow Region, 143025, Russian Federation

## Abstract

Telomerase is a key participant in the telomere length maintaining system in eukaryotic cells. Telomerase RNA and protein reverse transcriptase subunits are essential for the appearance of active telomerase *in vitro*. Telomerase is active in many cancer types and is a potential target for anticancer drug development. Here we report a new approach for impairing telomerase function at the stage of human telomerase assembly. The approach is based on the application of chimeric bifunctional oligonucleotides that contain two oligonucleotide parts complementary to the functional domains of telomerase RNA connected with non-nucleotide linkers in different orientations (5′-3′, 5′-5′ or 3′-3′). Such chimeras inhibited telomerase *in vitro* in the nM range, but were effective *in vivo* in sub-nM concentrations, predominantly due to their effect on telomerase assembly and dimerization.

## INTRODUCTION

Telomerase is a key participant in the telomere length maintenance system in the majority of eukaryotic cells that adds G-rich repeats to the telomere 3′-end. Two main components–the protein reverse transcriptase catalytic subunit (TERT) (1) and the telomerase RNA (TR) that encodes telomere repeats–are crucial for telomerase enzyme activity (2). A number of accessory proteins that facilitate telomerase assembly, access to telomere, activation, etc. are required for telomerase functioning *in vivo* (3). Telomerase is active in stem cells but not in normal tissues (4). About 85% of cancer cells use telomerase activation for telomere length maintenance and thus telomerase serves as a target for anticancer therapy (5,6). A number of functional domains were described for telomerase RNA. hTR contains the template for DNA synthesis and four other conserved domains found in vertebrate telomerase RNAs. The box H/ACA and CR7 domains are dispensable for reconstitution of telomerase activity *in vitro* (7) in contrast to two highly conserved structural domains: a template-proximal pseudoknot structure that forms a triple helix (8) and the three-way helical junction, referred as the CR4/CR5 domain (9). The latter domains were found to be crucial for telomerase function (10) and are present even in smallest vertebrate RNA (11). The pseudoknot and CR4/CR5 domains are essential for telomerase activity and bind independently to two distinct TERT domains (12–14).

Different strategies for targeting telomerase are available today, such as the use of small molecule inhibitors, antisense oligonucleotides, G-quadruplex stabilizers, inhibitors of telomere- and telomerase-associated proteins, immunotherapy and gene therapy (5,15). Among them, antisense oligonucleotides that interact with the hTR template and thus prevent interaction of telomerase with its natural substrate are already in the pre-clinical and clinical trials (Imetelstat) (16). Two examples of telomerase inhibition through telomerase assembly interference are described today: via affecting chaperones participating in this process (17) and via oligonucleotides that are complementary to hTR and inhibit telomerase activity in an *in vitro* reconstitution assay (18). Two oligonucleotides targeting the P3/P1 pairing region of the hTR (174–195) and CR4–CR5 domain (301–322) most effectively block the association between the telomerase catalytic subunit and hTR in partially reconstituted system.

Here we demonstrate the application of modified oligonucleotides to intervene in telomerase assembly. We synthesized chimeric oligonucleotides that contain two target sites that can simultaneously interact with two functional domains of hTR. The efficiency of the application of bifunctional oligonucleotides was recently proved for redirection of splicing in a variety of genes (19). In order to increase conformational flexibility, a non-nucleotide linker was introduced between the functional parts. Orientation (5′-3′, 5′-5′ or 3′-3′) of oligonucleotide parts within chimeras significantly influenced the inhibitory activity. The functional parts of chimeric oligonucleotides were complementary to either the template or single-stranded parts of a pseudoknot or the CR4/CR5 domain. We have shown that different chimeras used in this study inhibit either telomerase assembly or activity or both. The application of chimeras with two template-binding parts allowed us to interfere with hTR dimer appearance.

## MATERIALS AND METHODS

### Plasmids and oligonucleotides

Plasmids for transient transfection of human telomerase reverse transcriptase (hTERT) (p-DS-SFFV-hTERT) and hTR (pBluescript-DS-U1-hTR) have been documented previously (20). Non-modified oligonucleotides used for analysis were purchased from Syntol (Russia). Modified oligonucleotides were synthesized by the standard phosphoramidite method on an ABI 3400 DNA synthesizer (Applied Biosystems, USA) with minor changes: detrytilation was carried out with 6% dichloroacetic acid in 1,2-dichloroethane, coupling of all phosphoramidites was done with 4,5-dicyanoimidazole as an activator for 3 min, and capping was done with Cap A: Ac2O in THF and Cap B: DMAP/pyridine/THF followed by oxidation with 0.05 M iodine in THF/Py/water. Universal and C6-amino CPG (loading 40–50 μmol/g), common and 5′→3′-inverted 2′-OMe-U, 2′-OMe-A(Bz), 2′-OMe-G(iBu), 2′-OMe-C(Ac), c3 (1,3-propanediol), c9 (triethylene glycol) and HEG (hexaethylene glycol) linker phosphoramidites were purchased from ChemGenes (USA). After synthesis, solid supports were treated with AMA solution for 30 min at 65ºC. The supernatant was removed and the solid support was washed twice with AMA. The supernatant and washings were combined and the whole mixture was evaporated to dryness. Oligonucleotides were first purified by denaturing (7 M urea) PAGE, electroeluted from gel by Elutrap (Whatman) followed by RP-HPLC with an acetonitrile gradient in 0.05 M ammonium acetate (pH 7). HPLC purification was carried out on an ÄKTA Purifier (GE Healthcare, USA) equipped with a Jupiter C18 column (Phenomenex, Jupiter 5 μm, 300 Å, 250 × 4.6 mm) and a UV-Vis detector. Oligonucleotides after concentration were desalted by ethanol precipitation and characterized by mass spectrometry. MALDI MS spectra were recorded on an AutoFlex (Bruker Daltonics, USA) using 2,4,6-trihydroxyacetophenone/ammonium citrate, or 3-hydroxypicolinic acid/ammonium citrate, as a matrix. ESI MS spectra were recorded on q-TOF Maxis Impact (Bruker Daltonics, USA) equipped with a 1260 (Agilent) HPLC system using methanol gradient in 10 mM diisopropylamine/100 mM hexafluoroisopropanol (21).

### *In vitro* telomerase inhibition assay

#### Telomerase purification

For telomerase overexpression, Human Embryonic Kidney (HEK 293E) cells were grown in DMEM medium and transiently transfected with p-DS-SFFV-hTERT plus pBluescript-DS-U1-hTR (20). HEK293E cells in 10 cm Petri dish were transfected with 22 μg of plasmid DNA and Lipofectamine 2000 (Invitrogen) following the manufacturer's protocol. The mass ratio between hTERT- and hTR-expressing plasmids was 1:10 (2 μg p-DS-SFFV-hTERT and 20 μg pBluescript-DS-U1-hTR). After 2 days of post-transfection, cells were seeded to a T150 flask and incubated for 2 more days (episomal replication maintained plasmid concentration). Then cells were washed with PBS 1x, then trypsinized/washed. Cell pellets were resuspended in 10 ml of ice-cold CHAPS buffer (10 mM Tris-HCl, pH 7.5, 1 mM MgCl_2_, 1 mM EDTA, 0.1 mM phenylmethylsulfonyl fluoride, 5 mM b-mercaptoethanol, 1 mM DTT, 0.5% 3-[(3 cholamidopropyl)dimethylammonio]-1-propane sulfonate) and 10% glycerol, then incubated for 30 min at 4ºC. Then the extract was centrifugated at 16 000 × g for 30 min at 4ºC. The obtained S16 lysate was immediately processed further. The S16 lysate (in two tubes, 5 ml each) was centrifugated at 100 000 × g, on an MLA-80 rotor (Optima MAX-XP, Beckman Coulter), for 45 min at 4ºC through a 1 ml 20% glycerol cushion in a CHAPS buffer. The cushion is needed to reduce telomerase loss, since we had in the past observed up to 50% telomerase activity reduction due to obtaining S100 extract in the conventional way.

The resulting supernatant was layered on top of 1 ml CHAPS containing 20% glycerol in the centrifuge tubes, pre-blocked by 6 mg/ml BSA 2 h before centrifugation, then centrifuged at 210 000 × g, on an MLA-80 rotor for 2 h at 4ºC (Optima MAX-XP, Beckman Coulter). Then each cell pellet was dissolved in 1 ml TRAP 1x buffer (20 mM Tris-HCl (pH 8.3), 63 mM KCl, 1.5 mM MgCl_2_, 0.1 mg/ml BSA, 0.002% Tween-20, 1 mM EGTA) containing 10% glycerol, thereafter aliquoted, quick frozen in liquid nitrogen and stored at −80ºC. The purification factor for precipitation of telomerase was approximately 4 (Supplementary Table S1).

#### Cell-free RQ-TRAP

RQ-TRAP analysis was performed in two steps (20). To determine the interval of linear dependence of telomerase activity on partially purified telomerase extract, we used serial 2-fold dilutions of the extract (Supplementary Figure S1). This determination was carried out before each experiment. RQ-TRAP analyses were performed three times for each particular measurement and repeated independently two or more times for every oligonucleotide inhibitor. Oligonucleotide inhibitors were used in a range of 0.01 nM–10 μM. Model TSR8 oligonucleotide (5′-AATCCGTCGAGCAGAG(TTAGGG)7-TTAG) in a concentration range of 10 pM–10 attoM was used as a template in the RQ-PCR reaction to obtain a standard curve used for further quantitative calculations of telomerase activity level.

Five microliters of tested oligonucleotide in a 1x TRAP buffer with appropriate concentration was added to 5 μl of purified telomerase extract previously 50-fold diluted with a 1x TRAP buffer. The mixture was incubated for 10 min at room temperature, and 5 ml of ‘Mix 1’ (1x TRAP buffer, 0.3 μM TS (5′-aatccgtcgagcagagtt-3′), 0.3 μM dNTP) was added. The mixture was incubated at 30ºC for 30 min and inactivated by heating it to 80ºC for 10 min. After that 150 μl of a 1x TRAP buffer was added and mixed. To perform the PCR reaction, 5 μl diluted telomerase product was added to 5 μl of ‘Mix 2’ (1x TRAP buffer, 0.4 μM TS, 0.4 μM ACX (5′-cccttacccttacccttaccctta-3′), 0.4x SYBR Green I (Invitrogen), 0.2 mM dNTP, 0.1 U *Taq*-polymerase). Assembly of the PCR reaction was performed on ice. ‘Mix 2’ was prepared in a large amount, aliquoted and stored at −80ºC. Amplification was performed in on a 384-well PCR plate at CFX-384 Real-Time System, C1000 Thermal Cycler (Bio Rad), in 30 PCR cycles with 30 s at 95ºC, 30 s at 60ºC (plate reading) and 60 s at 72ºC. For Jc3N, 1500 μl of a 1x TRAP buffer, rather than 150 μl, was used after telomerase inactivation, before the PCR step described above.

The tested oligonucleotides were independently added both before and after telomerase extension in order to check inhibition of PCR. Determination of the Ct value was performed automatically by using a threshold line in Bio-Rad CFX Manager (version 1.6.541.1028), and all calculations and non-linear curve fittings were generated using GraphPad prism software.

#### Direct telomerase activity assay

Telomerase assays were performed as described previously (22) with 2 μl partially purified telomerase.

### *In vivo* telomerase inhibition test

Oligonucleotide transfection and cell extract isolation. HEK 293 cells were transfected by a oligonucleotide inhibitor with Lipofectamine 2000 (Invitrogen) according to the manufacturer's protocols with minor modifications. The oligonucleotide was serially diluted with the Opti-MEM®I Medium to concentrations of 220 μM, 22 μM, 2.2 μM, 220 nM, 22 nM, 2.2 nM, 0.22 nM, 0.022 nM. Subsequently, 5 μl of diluted oligonucleotide were mixed with 5 μl of 17.7-fold diluted Lipofectamine 2000 in 96-well plates. 2.5 × 10^4^ HEK293 cells diluted in 100 μl DMEM medium were added to each well after 15 min of incubation at room temperature. After 2 days of incubation at 37ºC in a humidified atmosphere of 5% CO_2_, cells were washed with PBS 1x, then lysed in 100 μl of CHAPS buffer for 30 min on ice. Cell debris was removed by centrifugation at 2464 × g for 30 min at 4ºC. Protein concentration was determined by the standard Bradford method and used for normalization of the extracts.

#### Cell-based RQ-TRAP

Cell extracts obtained from oligonucleotide transfection were diluted 10-fold with a 1x TRAP buffer. Then 5 μl of diluted extract were added to 5 μl of ‘Mix 2’ on ice. The telomerase reaction was performed at 10ºC for 1 h with subsequent PCR amplification the same as with the cell-free RQ-TRAP. It is known that *Taq*-polymerase is active in the range of 15–100ºC. We observed that telomerase is active even on ice, without a great decrease in product yield within 1–2 h. Therefore, 10ºC is enough to keep telomerase active and Taq-polymerase inactive. Amplification was performed on a 384-well PCR plate at a CFX-384 Real-Time System, C1000 Thermal Cycler (Bio Rad), in 30 PCR cycles for 30 s at 95ºC, 30 s at 60ºC (plate reading) and 60 s at 72ºC. Determination of Ct value was performed automatically by using a threshold line in Bio-Rad CFX Manager (version 1.6.541.1028). The empty buffer gave a signal in the range of 27–30 cycles. TSR8 oligonucleotide was used in the same way as the cell-free RQ-TRAP. To check linearity of the signal from the telomerase amount, extract from untransfected cells was 2-fold serially diluted.

A PCR inhibition test for certain oligonucleotides was performed by mixing 2.5 μl of 200-fold diluted partial purified telomerase with 2.5 μl of cell extracts obtained from oligonucleotide transfection and then diluted 10-fold with a 1x TRAP buffer. The mix was incubated for 10 min at room temperature. Five microliters of ‘Mix 2’ were added to the reaction mix on ice and amplification was performed as described above. A 2.5 μl of diluted purified telomerase mixed with 2.5 μl buffer was accepted as 100%.

### Telomerase assembly test

We used cells transfected with 5 μM of oligonucleotide inhibitors for telomerase assembly analysis. One hundred microliters of 5-fold diluted with 1x TRAP buffer cell extract (500 μl) was subjected to centrifugation in sucrose gradient (23). For that, the gradient was obtained by two times freeze-thaw cycle (−20ºC & RT) of 20% sucrose in 1x TRAP buffer (24). Centrifugation was made in SW-41 Ti rotor (Beckman) at 4°C for 20 h at 111 132 × g (30 000 rpm) on J2-HS Beckman centrifuge. Fractions of approximately 500 μl were collected starting from bottom. To detect telomerase activity, 2.5 μl aliquot from every fraction of gradient was used for cell-based RQ-TRAP reaction.

### hTR quantification by RQ-PCR

To each fraction, 2 μl of glycogen (20 mg/ml, Roche Applied Science) was added. Then RNA from each fraction was extracted with phenol:chloroform (1:1), precipitated with ethanol and dissolved in 50 μl mQ water. Five microliters of RNA isolated from every fraction of sucrose gradient and 1 μM hTR-R1 (5′-TGCTCTAGAATGAACGGTGGAA-3′) were used for reverse transcription. The reaction was performed in 15 μl volume at 50°C, 2 h by 0.25 μl Maxima Enzyme mix (Thermo Scientific) according to the manufacturer's protocols. Then 5 μl of 5-fold diluted reaction mixture proceeded to PCR in 25 μl containing 10 mM Tris-HCl (pH 8.8 at 25°C), 50 mM KCl, 0.08% (v/v) Nonidet P40, 0.1 mM dNTP, 0.2 μM hTR-F (5′-GTGGTGGCCATTTTTTGTCTAAC-3′), 0.2 μM hTR-R1, 1.5 mM MgCl_2_, 0.04 U *Taq-*polymerase, 0.25x SYBR-Green I at CFX-96 Real-Time System (Bio Rad), in 30 amplification cycles (30 s at 95°C, 30 s at 58°C and 60 s at 72°C). We used serial 10-fold dilution of *in vitro* synthesized hTR RNA as a standard. hTR was synthesized by T7-transcription (25) with minor modifications. Thus, template DNA for T7-transcription was obtained by PCR amplification (30 cycles: 40s 94°C, 20s 58°C, 90s 72°C) in 50 μl reaction mixture containing 1 ng plasmid pBluescript-DS-U1-hTR, 1× *Pfu* buffer (Fermentas), 0.5 μM T7-hTR-forward primer (5′-GGGGAAGCTTTAATACGACTCACTATAGGGTTGCGGAGGGTGGGCCTG-3′), 0.5 μM hTR-reverse primer (5′-CCCCGGATCCTGCGCATGTGTGAGCCGAGTCCTGGG-3′) and 0.2 mM dNTP, 0.1 U *Pfu*-polymerase. The PCR product was purified with a GeneJET PCR Purification Kit (Thermo Scientific). Then RNA obtained by a MEGAScript-T7 Kit (Invitrogen) was extracted with phenol:chloroform (1:1 v/v), precipitated with ethanol, purified by PAGE gel electrophoresis and quantified by ratio at 260/280 nm.

### hTR level in cell quantification by RQ-PCR

A 2.5 μl of cell extract that was obtained for the *in vivo* telomerase inhibition test were used for quantification of hTR levels in cells transfected with the oligonucleotide. Reverse transcription was performed as described above. We observed that the crude cell extract did not have effect on the reverse transcription reaction but did inhibit PCR. A 7-fold dilution of samples by mQ water eliminated the effect to PCR. Five microliters of diluted sample proceeded to PCR as described above. Untransfected cell extract was serially 2-fold diluted and used as a positive control to check linearity of signal from the cell extract amount. The relative expression level of RNA was calibrated by the geometric mean of GAPDH that was analyzed with primers Fw (5′-TGCACCACCAACTGCTTAGC-3′) and Rv (5′-GGCATGGACTGTGGTCATGAG-3′). All of the PCR amplifications were performed in triplicate.

### Cell viability (MTT) assay

Twenty microliters of 5 mg/ml MTT salt (in PBS 1x) was added to cells transfected and/or incubated 2 days with inhibitory oligonucleotides (see below). Then after 3-h incubation at 37ºC, 5% CO_2_ in humidified atmosphere medium was removed and 150 μl of DMSO was added and gently mixed. Samples were measured at 555 nm with a reference filter of 670 nm. Then the signal of the 670 nm filter was subtracted from the signal of the 555 nm. Obtained data were normalized to the signal from untransfected cells which were assumed as 100%.

## RESULTS

### Design of chimeric bifunctional oligonucleotides

**Figure 1.**
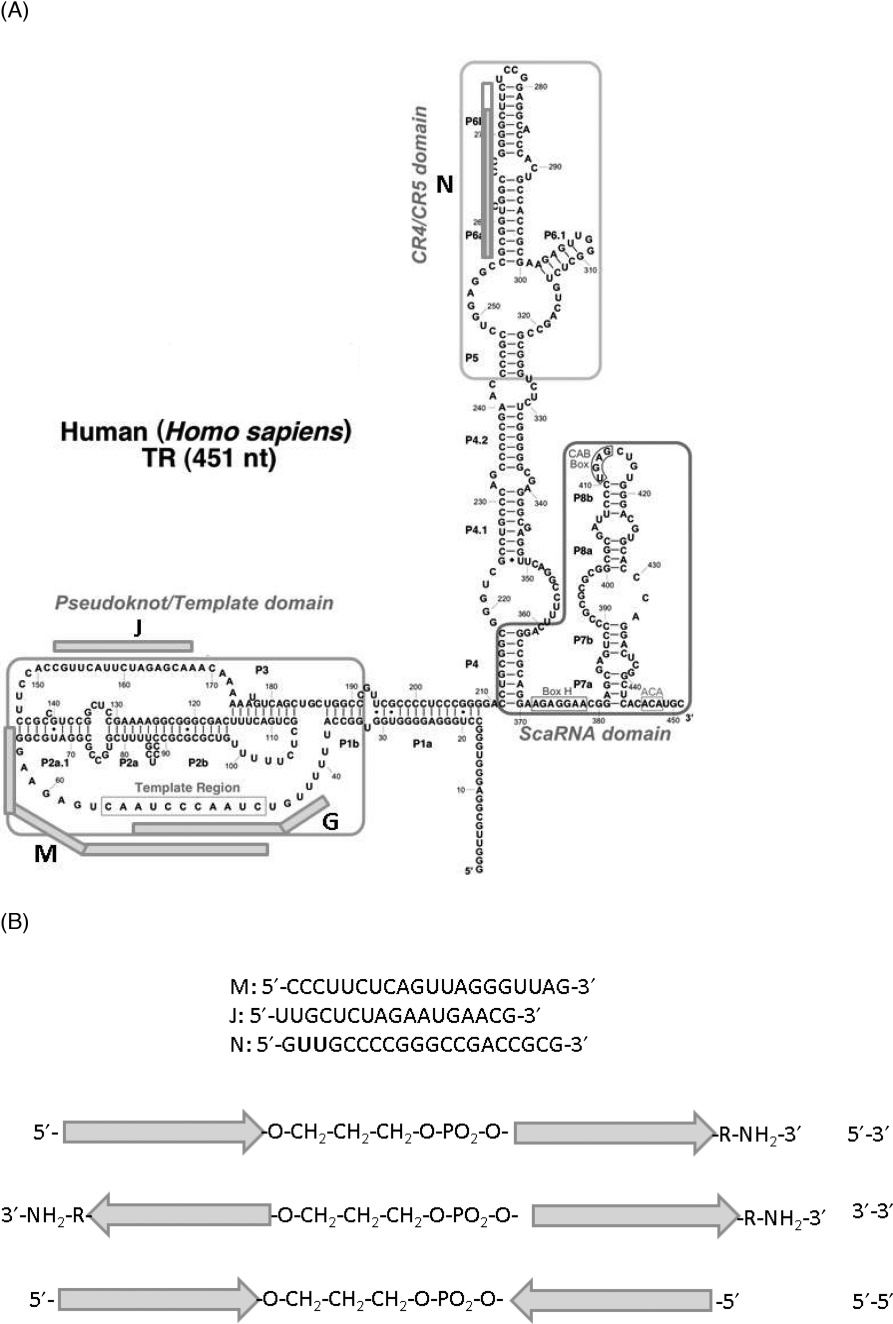
**(A)** Positions of oligonucleotide parts of chimeras in the hTR secondary structure (9). Oligonucleotides M, J, G and N marked with rods. The non-complementary oligonucleotide site for N is marked in white colour of the corresponding part of N rod. **(B)** Scheme of chimeras. Arrows correspond to 3′-end of M, J and N. Abbreviations corresponding to the orientation of oligonucleotide parts in the chimeras are indicated on the right.

We synthesized a number of chimeric oligonucleotides that comprised two parts connected with a non-nucleotide linker. These oligonucleotides (Table 1) contained the following parts: M–complementary to the template region (Figure 1A) (hTR position 46–65)–oligonucleotide analogs complementary to hTR within this region were shown to inhibit telomerase activity by interacting with the template (26); J (hTR position 152–168)–complementary to single-stranded chain of the pseudoknot (Figure 1A)–such oligonucleotide was shown to be telomerase inhibitor both *in vivo* and *in vitro* (20); N–contains complementary to CR4/CR5 region (Figure 1A)–the hTR position 256–272, region is known to be essential for telomerase assembly (10) and three additional nucleotides at the 5′ end that allow to separate complementary regions in chimeras when N is a part of either MN or JN chimeras. All oligonucleotides were modified with 2′-OMe nucleotides and connected together either via 3′-5′ or 3′-3′ or 5′-5′ ends with or without a 1,3-propane diol linker (c3) (Figure 1B). 3′-ends in all cases were blocked by addition of 6-aminohexanol. If M, N, J parts were used alone, they contained a c3 linker as a modification at the 5′-end. Oligonucleotide G (Figure 1A) (hTR position 42–54), with a sequence of a known telomerase inhibitor that binds to the hTR template (26), modified as described above, was used as a control.

**Table 1.** Inhibition of telomerase activity (IC_50_) by modified and chimeric oligonucleotides *in vitro* and *in vivo*

Oligonucleotides\ chimeras	Sequence of 2′-O-methyl oligonucleotides, position of complementary region of hTR	IC_50_, nM	
		*in vitro*	*in vivo*
c3N	C3-G**UU**GCCCCGGGCCGACCGCG-NH_2_ 272–256	No inhib.^b^	41.1 ± 7.4
c3M	C3-CCCUUCUCAGUUAGGGUUAG-NH_2_ 65–46	100 ± 20	5.2 ± 0.9
c3J	C3-UUGCUCUAGAAUGAACG-NH_2_ 168–152	19 ± 16 6.8 ± 0.8^a^	43 ± 21
c3G	C3-UAGGGUUAGACAA-NH_2_ 54–42	11.6 ± 5.6 5.5 ± 0.6^a^	67 ± 33
Mc3	CCCUUCUCAGUUAGGGUUAG-C3 65–46	75 ± 57 77 ± 10^a^	175 ± 137
Mc3N	CCCUUCUCAGUUAGGGUUAG-C3-G**UU**GCCCCGGGCCGACCGCG-NH_2_ 65–46&272–256	35 ± 18 118 ± 20^a^	2.2 ± 0.8
MNc3	CCCUUCUCAGUUAGGGUUAG-G**UU**GCCCCGGGCCGACCGCG-C3 65–46&272–256	No inhib.^b^	18.4 ± 5.6
c3MN	C3-CCCUUCUCAGUUAGGGUUAG-G**UU**GCCCCGGGCCGACCGCG-NH_2_ 65–46&272–256	No inhib.^b^	1.2 ± 0.4
M*c3N	(3′-NH_2_-GAUUGGGAUUGACUCUUCCC-5′)-C3- G**UU**GCCCCGGGCCGACCGCG-NH_2_ 46–65&272–256	15.7 ± 8.2 72 ± 8^a^	0.5 ± 0.1
Mc3N*	CCCUUCUCAGUUAGGGUUAG-C3-(3′-GCGCCAGCCGGGCCCCG**UU**G-5′) 65–46&256–272	13.5 ± 3.4	7 ± 1
Nc3M	G**UU**GCCCCGGGCCGACCGCG -C3- CCCUUCUCAGUUAGGGUUAG-NH_2_ 256–272&46–65	76 ± 21 27 ± 3^a^	1.3 ± 0.9
Nc3J	G**UU**GCCCCGGGCCGACCGCG -C3- UUGCUCUAGAAUGAACG-NH_2_ 272–256&168–152	No inhib.^b^	1.5 ± 0.7
N*c3J	(3′-NH_2_-GCGCCAGCCGGGCCCCG**UU**G-5′)-C3- UUGCUCUAGAAUGAACG-NH_2_ 256–272&168–152	No inhib.^b^	1.3 ± 0.6
Jc3N	UUGCUCUAGAAUGAACG –C3- G**UU**GCCCCGGGCCGACCGCG-NH_2_ 168–152&272–256	162 ± 23	3.9 ± 1.7
Nmisc3J	G**UUCG**CCCG**C**GCCGAC**GC**CG-C3- UUGCUCUAGAAUGAACG-NH_2_ 272–256&168–152	No inhib.^b^	8.5 ± 3.5
Nc3Jmis	G**UU**GCCCCGGGCCGACCGCG -C3-UUG**UC**CUA**AG**AUGA**CA**G-NH_2_ 272–256&168–152	No inhib.^b^	11.2 ± 0.8
Nmisc3Jmis	G**UUCG**CCCG**C**GCCGAC**GC**CG-C3-UUG**UC**CUA**AG**AUGA**CA**G-NH_2_ 272–256&168–152	No inhib.^b^	18.4 ± 3.2
compNc3J	CGCGGUCGGCCGCCGGCAAC-C3-CGUUCAUUCUAGAGCAA-NH_2_ 256–272(complem)& 152–168 (complem)	No inhib.^b^	no inhib.^b^
Mc3M	CCCUUCUCAGUUAGGGUUAG-C3-CCCUUCUCAGUUAGGGUUAG-NH_2_ 65–46&65–46	221 ± 10	1.4 ± 0.3
M*c3M	(3′-NH_2_-GAUUGGGAUUGACUCUUCCC-5′)-C3-CCCUUCUCAGUUAGGGUUAG-NH_2_ 46–65&65–46	11.9 ± 0.3 19.3 ± 2.5^a^	0.3 ± 0.1
MMc3	CCCUUCUCAGUUAGGGUUAGCCCUUCUCAGUUAGGGUUAG-C3 65–46–65–46	42 ± 1 26.7 ± 3.0^a^	4.5 ± 0.9
c3Mc3N	C3-CCCUUCUCAGUUAGGGUUAG-C3-GUUGCCCCGGGCCGACCGCG-NH_2_ 65–46&272–256	184 ± 58	0.7 ± 0.5
c3Mc3M	C3-CCCUUCUCAGUUAGGGUUAG-C3-CCCUUCUCAGUUAGGGUUAG-NH_2_ 65–46&65–46	44 ± 12	0.31 ± 0.04

^a^IC_50_ obtained from direct telomerase assay, in all other IC_50_ was calculated from RQ-TRAP.

^b^No inhibition; IC_50_ is more than 500 nM.

Non-complementary nucleotides marked in bold*.*

### Telomerase inhibition by chimeric oligonucleotides *in vitro*

To test telomerase activity *in vitro* we used a modified version of a classical RQ-TRAP, based on qPCR amplification of the DNA substrate elongated by telomerase. Telomerase was partially purified from a cell-free extract of HEK293E cells transfected with the plasmids overexpressing both hTR and hTERT. The first telomerase elongation reaction was carried out at the desired concentration of inhibitor followed by thermoinactivation of telomerase. Then the reaction mixture was diluted to exclude the effect of inhibitor at the PCR step. In each case, the absence of inhibitor influence on amplification was tested in a separate experiment. The data obtained for G oligonucleotide were used for standardization of the system. Examples of original graphs for IC_50_ calculation (concentrations resulting in 50% inhibition of telomerase activity) are presented in Supplementary Figure S2. The data are summarized in Table 1.

**Figure 2.**
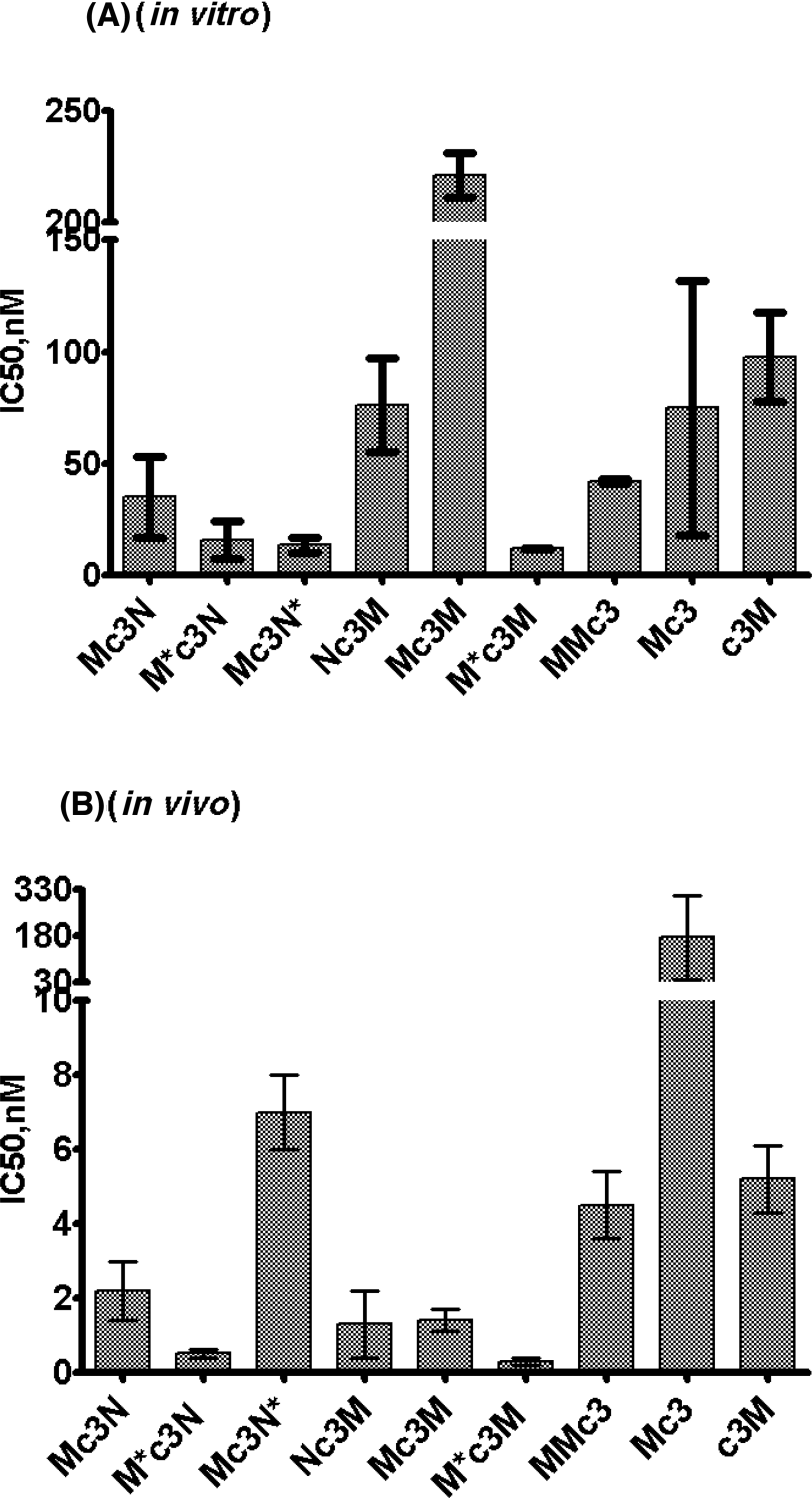
Inhibition of telomerase by M-containing modified oligonucleotide and chimeras. **(A)***in vitro* and **(B)***in vivo*.

M and J oligonucleotides alone showed a pronounced inhibitory effect on telomerase activity *in vitro* (IC_50_ 100 nM and 19 nM, correspondingly) (Table 1). In case of M oligonucleotides, the position of c3 modification either at 5′- or 3′-end did not influence the inhibition (Figure 2A). N oligonucleotide did not show any detectable inhibitory effect *in vitro*. G oligonucleotide showed better inhibitory properties than corresponding M ones (IC_50_ 11nM) (Table 1). For the 5′-3′ chimera Mc3N, the inhibitory effect was slightly better than for M alone (IC_50_ 35 nM). However, when M and N were connected within a long oligonucleotide with c3 moved to the 3′-end (MNc3), no inhibitory effect was detected. In the case of the 5′-5′ chimera M*c3N (inverted M) and 3′-3′ chimera Mc3N* (inverted N) inhibitory activity increased approximately two times in comparison to Mc3N. For Nc3M chimera, inhibitory activity decreased (IC_50_ 76 nM) (Figure 2A). In the case of J containing chimeras the inhibitory activity significantly (two times) reduced for Jc3N in comparison with J alone and was scarcely detectable in inverted forms. Mc3M showed very little inhibitory effect, although M*c3M (first M inverted) showed an IC_50_ value 11.9 nM significantly (7-fold) higher than M alone, and IC_50_ value of MMc3 was similar to the one for Mc3 (Figure 2A).

A direct telomerase assay (22) was used to verify the data obtained by RQ-TRAP for several oligonucleotides. The data are shown in Supplementary Figure S3 and calculated IC_50_ values are presented in Table 1. The IC_50_ obtained by both methods were in agreement.

### Inhibition of telomerase activity by chimeric oligonucleotides *in vivo*

Since parts of chimeras may intervene in telomerase assembly, we tested their influence on telomerase activity *in vivo*. HEK293 cells were cultivated in the presence of oligonucleotides transfected in a desirable concentration, and telomerase activity was determined in cell lysates by RQ-TRAP. The data are shown in Table 1. Examples of the original graphs for IC_50_ calculation are presented in Supplementary Figure S4.

In the case of G oligonucleotide, we observed a decrease of inhibitory effect (IC_50_ 67 nM) in comparison with the *in vitro* test. In case of M oligonucleotide with c3 at 5′-end, the inhibitory effect (IC_50_ 5.2 nM) was one order of magnitude higher then *in vitro*. When c3 was attached to the 3′-end of M, IC_50_ increased significantly (175 nM) in contrast to the *in vitro* system where such changes in c3 position had a marginal effect (Figure 2B). In the case of N oligonucleotide, a significant inhibitory effect (IC_50_ 41 nM) was detected, although N did not inhibit telomerase *in vitro*. Additionally, J oligonucleotide demonstrated IC_50_ value similar to N.

The most pronounced differences in IC_50_ values were observed in chimeras. For all chimeras these values were several times lower than the one for G and corresponding values *in vitro*. Chimera Mc3N showed IC_50_ 2.2 nM, while 5′-5′ inverted chimera M*c3N demonstrated even 4 times lower IC_50_ (0.5 nM), which demonstrated to be one of the most effective of all of the oligonucleotides in this study. 3′-3′ Mc3N* demonstrated IC_50_ value 3 times higher than the original 5′-3′. Nc3M demonstrated an effect similar to the original chimera.

To investigate the linker length effect on the inhibitory properties of Mc3N chimeras we synthesized oligonucleotides connected with triethylene and hexaethylene glycol. MNc3 was used as a zero length linker. The results are shown in Supplementary Figure S5. The data indicate that the length of the linker does not influence the inhibitory effect; however, the presence of any linker between the parts of chimera reduced the IC_50_ value by ten times. Since the position of the linker was essential for M inhibitor *in vivo* we tested c3MN (c3 at 5′-end). The IC_50_ value (1.2 nM) became comparable with IC_50_ for Mc3N. The c3Mc3N chimera demonstrated a 2-fold increase in inhibitory effect (IC_50_ 0.7 nM). Nc3J chimeras demonstrated an effect similar to Mc3N, while inversions of oligonucleotide parts did not result in significant changes in IC_50_.

To support that such strong inhibitory effects from chimeras were due to complementary interactions, mismatches were introduced into each part of Nc3J chimera (Nmisc3G, Nc3Jmis, Nmisc3Jmis) (Table 1). In all cases, this resulted in 6–10-fold increase in IC_50_ values. Substitution of N and J in Nc3J chimera for complementary ones (compNc3compJ) (Table 1) resulted in complete loss of telomerase inhibition.

Mc3M chimera, which was not very efficient in telomerase inhibition *in vitro*, revealed a high inhibitory effect *in vivo* (IC_50_ 1.4 nM). c3Mc3M in the inhibitory effect and 5′-5′ chimera M*c3M (first M inverted) demonstrated even lower IC_50_ 0.3 nM, which was the best among studied oligonucleotides. MMc3 also demonstrated a significant level of inhibition (4.5 nM), although it was three times less efficient than chimera with a linker inside (Figure 2B).

We have shown that transfection by c3N, c3M, c3J and chimeras Nc3J, M*c3M, Jc3N had no specific effect on PCR step of cell-based RQ-TRAP (Supplementary Table S2).

To test the effects from chimeras beyond telomerase, an MTT test for the most effective inhibitors (M*c3M, c3MN, Mc3N, Nc3J, M*c3N, N*c3J) was performed (Supplementary Figure S6). None of the chimera was distinctly toxic for the cells, thus suggesting little effect of chimeras for the processes beyond telomerase inhibition.

### Telomerase RNA level *in vivo* in the presence of chimeric oligonucleotides

The amount of hTR in the cell lines transfected with different chimeras at various concentrations was measured by qRT-PCR with GAPDH mRNA as an internal standard. Chimeric oligonucleotides did not noticeably influence hTR stability up to high concentrations (100 nM) of oligonucleotide (Supplementary Figure S7).

### hTR incorporation into telomerase complex in presence of chimeric oligonucleotides *in vivo*

**Figure 3.**
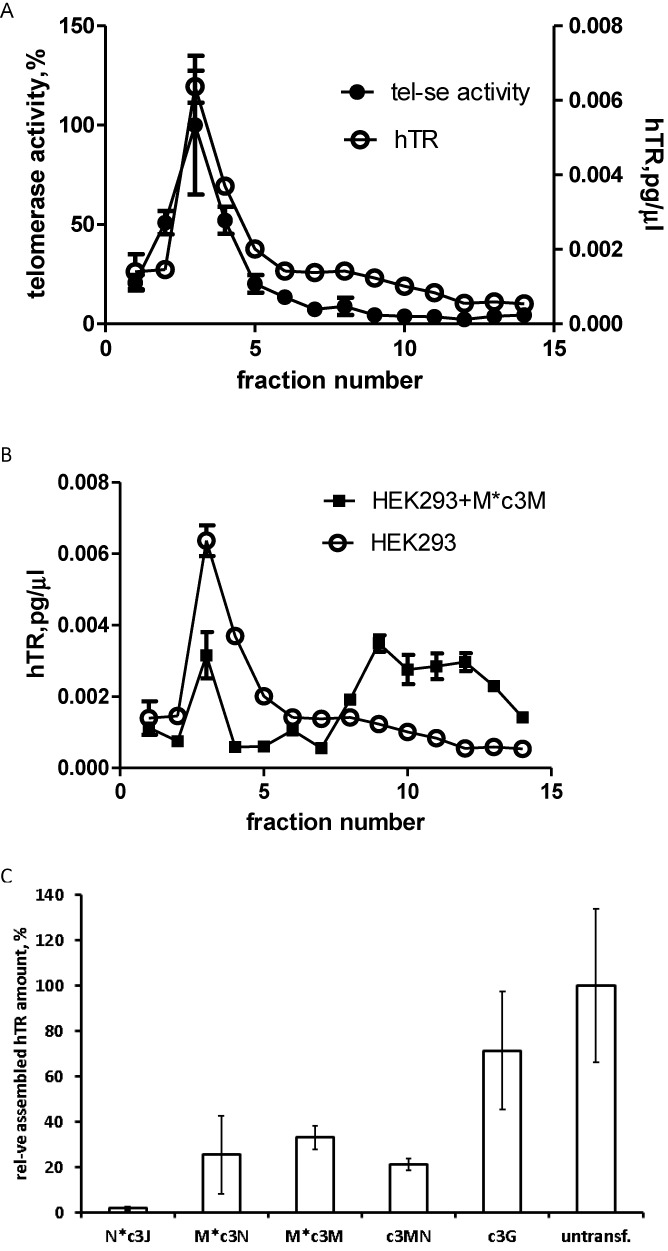
Amount of hTR incorporated into telomerase complex. **(A)** Distribution of hTR (○) and telomerase activity (•) after separation of HEK 293 cell-free extract in sucrose gradient. **(B)** Comparison of hTR distribution after separation of HEK293 cell-free extract (○) and cell-free extract at the presence of M*c3M chimera (▪). **(C)** The estimated assembly level of telomerase complex in the presence of chimeric oligonucleotides *in vivo*.

To test whether chimeras interfere telomerase assembly fractionation gradient was done on a discontinuous sucrose. Extracts obtained from cells transfected with a corresponding chimeric oligonucleotide at a concentration that exceeds IC_50_ were subjected to sucrose gradient centrifugation to separate assembled telomerase complexes from free hTR. Fractions were collected and the amounts of telomerase RNA and telomerase activity were measured. The data are shown in Figure 3. In HEK293 cells observed peak for hTR amount correlates with the peak of telomerase activity (Figure 3A) thus proving that this peak corresponds to assembled telomerase. The comparison of hTR distribution for hTR in HEK293 cells and cells with M*c3M chimera (the most effective telomerase inhibitor *in vivo*) shown in Figure 3B reveals a significant drop in the hTR (22% remained) in assembled telomerase peak. The data for c3J and other efficient chimeras Nc3J, M*c3N, c3MN are shown in Supplementary Figure S8 and summarized in Figure 3C. All most active chimeras significantly affected telomerase assembly. The strongest effect was observed in the case of Nc3J, where almost no assembled telomerase complex was detected, and significant loss of assembled complex was detected for all other chimeras. It should be mentioned that c3G only slightly interfered telomerase complex formations (Figure 3C).

## DISCUSSION

Until today, most effective antisense telomerase inhibitors were designed to bind to hTR and to inhibit already assembled enzymes. Most popular inhibitors are oligonucleotide derivatives that bind to the template region of hTR (27). Another region of hTR, which allows oligonucleotide binding to the assembled telomerase, is the single-stranded arm of the hTR pseudoknot (20). Here we designed bifunctional oligonucleotides that can interfere with both telomerase assembly and telomerase activity. These chimeras comprised two parts: the first part can bind either to the template region (M, Figure 1, Table 1) or the pseudoknot (J, Figure 1, Table 1) of hTR secondary structure model (9) and second part (N, Figure 1, Table 1) can bind to CR4/CR5 region of hTR known to be involved in interaction with hTERT (10). N was supposed to interfere telomerase assembly but did not influence telomerase activity. A non-nucleotide c3 linker (Figure 1) was used to separate functional parts in chimeras. In the case of chimeras with N as a second part, the linker between functional parts of chimera was more complicated and comprised a c3 linker and 3 nucleotides at the 5′-end of N that were not complementary to hTR (Figure 1). Three nucleotides non-complementary to hTR were introduced to provide additional flexibility between two oligonucleotide parts in MN and JN chimeras. To avoid degradation, all nucleotides were 2’-OMe modified and 3′-end of chimera was blocked with 6-aminohexanol to prevent both degradation by nucleases and elongation by polymerases. Telomerase inhibitory activity of M, J, N alone and as a part of the chimeras was determined both *in vitro* and *in vivo* (Table 1).

As we expected, M and J were able to inhibit telomerase *in vitro* alone, J was slightly better than M, while N alone did not inhibit telomerase. When M was combined into chimeras, the inhibitory effect for Mc3N slightly increased. Such an increase can be explained by a possible co-operative effect. Indeed, for MNc3 in which the functional parts are combined in one oligonucleotide with c3 attached to the 3′-end, the inhibitory activity was abolished, suggesting that the additional linker and conformational flexibility are essential to avoid steric problems in telomerase catalytic cavity and to allow proper binding of chimera to telomerase complex *in vitro*. Inhibitory effects almost disappeared in the case of Jc3N. Inversion of the J part (5′-5′ junction) or changing the order of the parts (Nc3J) did not restore inhibitory effect. Such effects could be explained by steric difficulties for the interaction of a large and flexible object with the pseudoknot arm in assembled telomerase.

A completely different situation was observed *in vivo*. Even application of an N part alone showed significant telomerase inhibition (IC_50_ 40 nM). Combinations of N with M or J (in all M-N and J-N chimeras) provided 10- to 100-fold increase of inhibitory activity. Such a difference in inhibitory activity of chimeras *in vitro* and *in vivo* allowed us to suggest that chimeras could interfere telomerase assembly. Imetelstat, a drug currently undergoing clinical trials, contains a number of additional modifications specifically targeted to increase its stability and cell permeation (28–30). The chimeras in this study, in the best test cases, were more than 100-fold more effective inhibitors than Imetelstat's G inhibitors, which have the sequence GRN163 with the same modifications as used here in chimeras. Therefore, the inhibitory properties of chimeras potentially could be further improved by additional chemical modifications described for GRN163 (29).

To determine that complementary interactions are indeed the basis for inhibition, in the Nc3J chimera mismatches were introduced to either the N or J moiety or both. That resulted in significant reduction of the inhibitory effect. Chimera with parts non-complementary to hTR (compNc3compJ) did not inhibit telomerase *in vivo*. That provides evidence that the inhibitory effect *in vivo* is based on specific complementary interactions of oligonucleotide parts of a chimera with corresponding regions of hTR.

J and N containing chimeras were shown to be efficient inhibitors; in these chimeras the inhibitory properties depended on the order of functional parts but not on their inversions (Table 1), indicating that the flexibility, linker size and proper orientation of functional parts may be essential for interactions with free hTR. Similar to J and N chimeras, chimeras with M and N changing the order of the functional parts also increased their inhibitory effect. However the most significant increase in inhibitory activity was observed for the chimera with inverted M (M*c3N) (Table 1). Such an increase can indicate that mutual orientation of oligonucleotide parts could be essential for interaction with hTR. On the other hand, we noticed that in the case of M, the modification at the 5′-end had significant effect *in vivo*. That may happen either due to the interference of c3 at the 3′-end with hTR interactions or that modification at the 5′-end may protect M from degradation. To demonstrate that, we used a c3Mc3N chimera in which the 5′-end was additionally protected. In the case of this chimera, the inhibitory effect increased, but not as much as in the case of the chimera with inverted M. Thus, the increase in the inhibitory effect of M*c3N may be explained by M stabilization at both ends, as well as the more favorable orientations of the functional parts of the chimera. It should be mentioned that the effect of stabilization due to modification at the 5′-end was specific to M. Indeed, N, Nc3J and N*c3J (the last having protection at both ends) had almost the same IC_50_.

Both MN- and NJ-containing chimeras had a very high inhibitory effect *in vivo* but not *in vitro*. That indicates that this effect might be due to impaired telomerase assembly. We gathered additional experimental evidence that the most effective chimeras indeed diminish the amount of assembled telomerase complex (Figure 3C, Supplementary Figure S8). JN-containing chimeras blocked telomerase assembly almost completely (Figure 3C) and MN-containing chimeras diminish the amount of assembled telomerase 5-fold (Figure 3C). Thus, for NJ-containing chimeras telomerase assembly is the main target of inhibition. In the case of MN-containing chimeras, we can propose that the inhibition involves both telomerase assembly and telomerase enzyme.

Unexpected data were obtained for MM-containing chimeras. M is complementary to the template region of hTR, and as in the case of GRN163 (29), its inhibitory effect should occur due to the interfering telomerase enzyme function.

*In vitro*, Mc3M inhibitory activity became significantly lower in comparison with Mc3 (or c3M) alone, similar to the effect of the fusion of either M or J with N (Jc3N and Mc3N). However, in contrast to other cases, the inversion of M in the chimera (M*c3M) resulted in a 20-fold increase of inhibitory effect *in vitro*, and the inhibitory effect of this chimera was better than that of MMc3. We propose that MMc3 can block one of the subunits of telomerase dimer similar to G; in Mc3M chimera, the appearance of conformational mobility somehow prevents its efficient binding to telomerase complex as in other chimeras, but in the inverted version M*c3M, changes in the orientation of oligonucleotide parts make it possible to interact with both hTR templates in the telomerase dimer (31). Low-resolution structure of the dimer was determined by cryoEM (32). Possible template positions proposed in the telomerase dimer structural model suggest that, in M*c3M chimera, the positions of oligonucleotides are more favorable for interactions with hTR templates in a dimer.

*In vivo*, similar to previously described cases, all chimeras connecting M and M showed significantly higher inhibitory activity (Figure 2B). For the Mc3M chimera, it increased five times than for the c3M. Chimeras with the inverted M (M*c3M) showed a further 5-fold increase of inhibitory activity, exhibiting the best IC_50_ (0.3 nM) among all oligonucleotides investigated, implying that these chimeras may also interfere with telomerase assembly. Experimental data on the amount of telomerase RNA incorporated in a telomerase complex revealed that G oligonucleotide complementary to the template only slightly reduced telomerase assembly (Figure 3C). This effect became very strong for the M*c3M chimera, which was comparable with that one Mc3N chimera that incorporates a moiety (N domain) to prevent telomerase assembly. These findings can be explained by disruption of an already-assembled complex that takes place in the cell more efficiently than in the case of an isolated telomerase. However, the more likely supposition is that chimeras with two parts complementary to the hTR template region can interact with two hTR templates in a dimer that forms at the early stages of telomerase assembly, thus impairing further telomerase assembly events. Indeed, *in vivo*, all M and M-containing chimeras were demonstrated as efficient inhibitors. They inhibited telomerase activity significantly better than ‘single M,’ indicating that both M moieties are important for inhibition. The lack of assembled telomerase in the case of M*c3M chimeras (Figure 3C) allows us to propose that chimeras interact with free hTR, while the necessity of two M moieties suggests the free hTR dimer participates in the interaction with M and M-containing chimeras. We posit that the difference in IC_50_ for such chimeras *in vivo* are less pronounced than *in vitro* are most probably due to increased flexibility of hTR domains in free hTR, in comparison to hTR in an assembled telomerase complex. The highest inhibitory activity of the M*c3M chimera can thus be explained by its increased stability in cells. Indeed, c3Mc3M chimera stabilized at the 5′-end demonstrated the same IC_50_ value. Thus, in the case of MM chimeras, the high inhibitory effect could be attributed to both impairment of telomerase assembly and inhibition of telomerase enzyme.

It should be mentioned that M*c3M chimeras with the unusual 3′-3′ orientation of the oligonucleotide parts was one of the most efficient inhibitors *in vivo* (Table 1). Although the synthesis of such compounds has been described in literature (33,34), there are very few examples of the application of such oligonucleotide chimeras for functional studies (35,36). In this study, we have demonstrated that such chimeras could be efficient tools to study functional properties of RNA containing complexes.

We have shown that the oligonucleotide chimeras, in which functional parts which interact with functional domains of hTR connected with non-nucleotide linker, can be efficient telomerase inhibitors that interfere both with telomerase assembly and telomerase enzyme functions. The application of chimeras with two template-binding parts allowed us to interfere with hTR dimer appearance.

Chimeric bifunctional oligonucleotides could be considered as a basis for the development of new, safer anti-cancer drugs. NJ containing chimeras have the advantage of not containing a sequence of telomere repeats that have, up until now, shown to affect additional non-telomerase targets in living cells.

## SUPPLEMENTARY DATA

Supplementary Data are available at NAR Online.
